# A pilot randomized trial examining the feasibility and acceptability of a culturally tailored and adherence-enhancing intervention for Latino smokers in the U.S.

**DOI:** 10.1371/journal.pone.0210323

**Published:** 2019-01-11

**Authors:** Marcel A. de Dios, Miguel Ángel Cano, Ellen L. Vaughan, Sarah D. Childress, Morgan M. McNeel, Laura M. Harvey, Raymond S. Niaura

**Affiliations:** 1 Department of Psychological, Health, and Learning Sciences, College of Education, University of Houston, Houston, Texas, United States of America; 2 Health Research Institute, University of Houston, Houston, Texas, United States of America; 3 Department of Epidemiology, College of Public Health and Social Work, Florida International University, Miami, Florida, United States of America; 4 Department of Counseling and Educational Psychology, School of Education, Indiana University, Bloomington, Indiana, United States of America; 5 Department of Social and Behavioral Sciences, College of Global Public Health, New York University, New York, New York, United States of America; University College London, UNITED KINGDOM

## Abstract

Latino smokers in the United States (US) are known to experience smoking cessation treatment disparities due to their under-utilization of services, limited access to health care, and poor smoking cessation treatment outcomes. A limited number of studies have focused on developing and testing smoking cessation treatments for Latino smokers in the US. The objectives of this study were to conduct a feasibility pilot randomized trial testing three smoking cessation interventions for Latinos. Twenty-five adult Latino smokers were randomized to one of three groups: Culturally-Tailored Smoking Cessation plus Adherence Enhancement (CT+AE), Culturally-Tailored Smoking Cessation (CTSC), and a Health Education (HE) control group. All participants received three counseling sessions along with nicotine replacement therapy (NRT). Data relating to intervention acceptability and NRT adherence were collected. Self-reported 7-day point prevalence smoking was collected at 3 and 6 month follow-up and biochemically verified with expired carbon monoxide testing. Overall, the interventions demonstrated high levels of feasibility and acceptability. Days of nicotine patch use were found to be higher in the CT+AE group (mean (*M)* = 81.3;standard deviation (*SD)* = 3.32) than the CTSC (*M* = 68.6;*SD* = 13.66) and HE (*M* = 64;*SD* = 17.70) groups. At 3-month follow-up, approximately 50% of the CT+AE group were smoking abstinent, 25% of the CTSC group, and 44% of the HE group. At 6-month follow-up, 37.5% of the CT+AE group were abstinent, 25% of the CTSC group, and 44.4% of the HE group. This study is the first to target Latino smokers in the US with a culturally-tailored intervention that addresses treatment adherence. Results support the preliminary feasibility and acceptability of the CT+AE intervention.

**Trial Registration**: ClinicalTrials.gov NCT02596711.

## Introduction

Approximately 15.5% of the adult population (38.7 million) in the United States (US) smokes tobacco [1]. It is the leading cause of preventable disease and death with over 480,000 deaths attributed to smoking each year [1]. Smoking has been identified as a critical public health problem among Latinos. A 2014 national survey of Latino health in the US found an overall daily and non-daily smoking prevalence rate of 26.1% among Latino males and 16.4% among Latina females, with substantial disparities by both gender and national origin [2]. For example, smoking among Mexican American males (23.4%), Cuban American males (31.3%) and females (21.9%), as well as Puerto Rican males (35%) and females (32.6%) exceeds the smoking prevalence rate of non-Latino Whites (19.4%) [2]. Among Latinos in the US, which are estimated to be 18% (~58 million individuals) of the US population [3], three out of the four leading causes of death (cancer, heart disease, and stroke) are associated with smoking [4]. Moreover, lung cancer is the foremost cause of cancer death for Latino men and the second leading cause of death for Latina women [4].

Latino smokers also experience smoking cessation treatment disparities due to a general lack of health care access [5–8], under-utilization of existing services [9], and poor treatment outcomes [10–12]. Results from various national surveys indicate that Latinos receive advice to quit smoking from their physician at lower rates than other ethnic/racial groups [13–15]. In multiethnic/ racial treatment studies, Latinos have been shown to have lower smoking cessation abstinence rates as compared to Whites [11, 12, 16].

A relatively limited number of studies have tested smoking cessation interventions with Latino smokers [17]. Of the existing studies, only five utilized a randomized controlled trial (RCT) design that included a control group and biochemical measures of smoking abstinence at follow-up [18–22]. In Webb and colleague’s (2010) meta-analysis that included these five studies, an odds ratio effect size (ES_OR_) of 1.54 (95% confidence interval (CF), 1.09–2.16) was calculated, indicating that the experimental treatments in the selected studies had a significant effect on cessation rates. However, this effect was only found at the *end of treatment* and deteriorated by follow-up [17]. Since that review, Cabriales and colleagues [23] found increased rates of abstinence among their intervention group at the end of treatment, but not through to follow-up. The most recent RCT [24] tested a smoking cessation intervention for the partners of pregnant Latinas. Participants showed promising rates of abstinence at the conclusion of treatment, yet there were no differences at follow-up as compared to the control group [24]. These findings highlight both the challenge of developing smoking cessation treatments for Latinos and the continued need for treatment development studies for this population.

It has been 10 years since the U.S. Department of Health and Human Services published its updated *Clinical Practice Guideline for Treating Tobacco Use and Dependence* [25] which urged the advancement of research focusing on the development and testing of culturally adaptive smoking cessation for racial minority populations including Latinos [25]. Since that time, there have been a limited number of published randomized controlled trials of smoking cessation interventions specifically tailored to Latinos [24, 26–28]. Considering the lack of research attention and the disparities in smoking cessation among Latinos, further research is needed.

Decades of research support the efficacy of various smoking cessation pharmacological treatments, such as nicotine replacement therapy (NRT), for the general population [29, 30]. In fact, NRT is the most widely used and available smoking cessation pharmacotherapy, and it has been found to increase the likelihood of quitting by up to two-fold [31–33]. Yet, Latino smokers exhibit a general disinclination towards using smoking cessation pharmacotherapies, prompting investigators to explore the unique characteristics of Latino smokers that may contribute to this phenomenon [34, 35].

Levinson and colleagues [34] identified potential barriers to Latino smokers’ use of pharmacotherapies for cessation: concerns about side effects, fears of becoming dependent on medications, cultural inclinations towards quitting without chemical aid, a lack of knowledge about the effectiveness and use of medications, and misconceptions about the perceived risks of smoking. Not surprisingly, adherence to pharmacotherapy has been identified as a significant barrier to smoking cessation success among Latinos [27, 36]. When Latino smokers make use of smoking cessation pharmacotherapies, they utilize them at a considerably lower rate than the minimum practice guidelines [25].

The current study extends this line of research and pilot tests a culturally-tailored smoking cessation intervention (CTSC), as well as an adherence enhancement “add-on” intervention (CT+AE). This study compared three “active” and dosage-matched interventions and included biochemical-verification of smoking abstinence at 3- and 6-month follow-up. We hypothesized that (a) both the CTSC and CT+AE groups would demonstrate intervention acceptability among our target population and feasibility within the context of an urban medical center clinic. (b) the CT+AE group would show greater levels of NRT patch usage as compared to the CTSC and HE groups and (c) the CTSC and CT+AE groups would have greater rates of biochemically verified 7-day point prevalence smoking abstinence at the 3- and 6-month follow-up visits.

## Methods

### Participants

The current study was a 3-group feasibility pilot randomized trial of 25 Latino smokers. All participants received 12 weeks of nicotine patch treatment and self-help materials. Participants were recruited from the Houston metropolitan area (Harris County, Texas) through fliers at community centers and local health clinics as well as through advertisements online and in print newspapers. Latinos constitute 43.3% (~2 million) of the population of Harris County [37] and smoking prevalence rates are estimated to be within the range seen nationally [38].

Study personnel screened a total of 98 individuals by phone between February 2016 and January 2017 using the study eligibility criteria. The following inclusion criteria were used: (a) 18+ years of age; (b) current smoker of at least five cigarettes per day, for the past 3 months; (c) able to speak and read English or Spanish; (d) agree to participate in the study; (e) available for study visits including the 3-month and 6-month follow-up assessment visits; (f) willing to set a quit date within 2 weeks of enrolment; and (g) identify as being of Latino heritage, ethnicity, or ancestry. Exclusion criteria included individuals: (a) suffering from any unstable medical condition precluding the use of NRT; (b) currently using smokeless tobacco, electronic nicotine delivery systems (ENDS), NRT, or other smoking cessation treatment; (c) pregnant or nursing; (d) suffering from a severe psychiatric disorder that would interfere with participation; (e) diagnosed with any substance dependence disorder other than nicotine and screened with the Diagnostic and Statistical Manual of Mental Disorders 4th Edition, Text Revised (DSM-IV-TR) [39], and (f) no access to a working telephone. Recruitment and enrollment were conducted during March of 2016 to January of 2017. A total of 53 eligible individuals were scheduled for the baseline study visit at the University of Texas MD Anderson Cancer Center. The remaining 45 individuals were ineligible; the most common reasons for ineligibility were unwillingness or inability to schedule the baseline visit (n = 12), subthreshold cigarettes smoked per day (< 5; *n* = 8) and heart problems precluding the use of NRT (*n* = 6).

**Fig 1:**
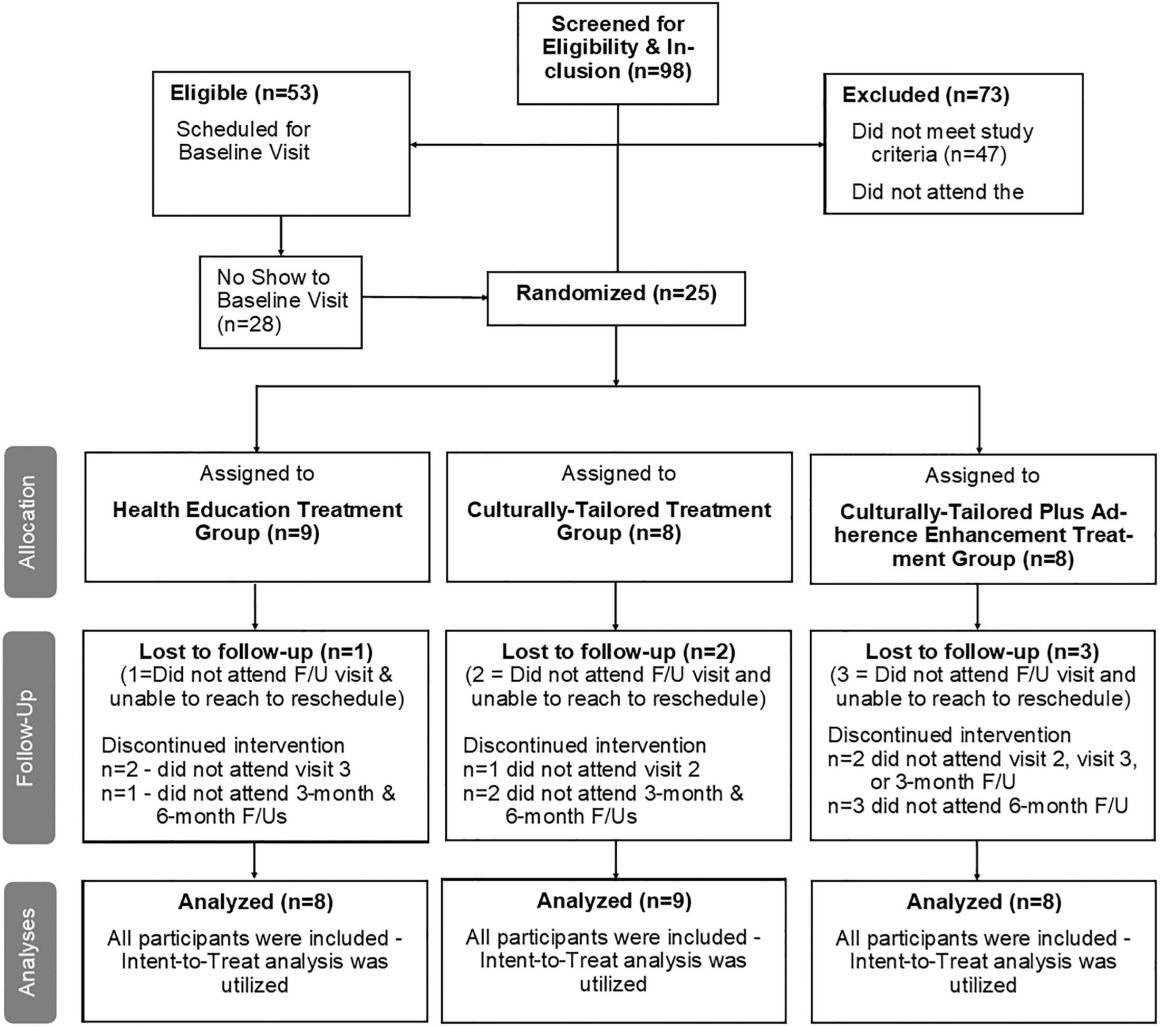
Participant flow diagram.

Of the 53 eligible individuals, 26 did not attend the Baseline visit and were not enrolled in the study (Fig 1). Data for these individuals (including demographic characteristics and reasons for missing the visit) are not available. Two additional individuals were removed from the study for no longer meeting inclusion/exclusion criteria at the baseline visit. The final study sample consisted of 25 participants. At the baseline appointment, participants underwent randomization to one of three treatment groups: (a) Health Education (HE, *n* = 9), (b) Culturally-Tailored (CTSC, *n* = 8), or (c) Culturally-Tailored plus Adherence Enhancement (CT+AE, *n* = 8) using an urn randomization technique generated by the Principal Investigator (1:1:1) and stratified by gender. Sealed envelopes containing treatment assignments were prepared prior to the commencement of the trial. Randomization envelopes were kept locked in the Principal Investigator’s (PI) office and each envelope was given to the assigned interventionist on the day of the first session with participants. The envelopes were opened after receiving confirmation that the participant arrived, met eligibility criteria, and provided their consent to participate in the trial.

### Study visits

Participants randomized to each of the three treatment intervention groups received three manualized individual counseling sessions. The general duration of these sessions and their schedule (at baseline, and 2 & 6 weeks post-baseline) was the same across all conditions. All participants received 12 weeks of NRT patch treatment—Nicoderm CQ [40], which they were advised to use according to the standard tapering schedule [27].

During the baseline appointment, participants completed a battery of assessments and participated in a 45- to 60-minute counseling session. During this session, participants worked with the interventionist to set a quit date within 2 weeks. Participants returned at 2 and 6 weeks post-baseline to complete a short assessment and have a counseling session. Patch dispensation also occurred at each of the study visits and included self-help brochures and materials, which consisted of the consumer products developed for the *Treating Tobacco Use and Dependence Clinical Practice Guidelines* [41]. After the Week 6 visit, participants were scheduled to return for 3- and 6-month follow-up visits, which occurred between June 2016 and June 2017. A trained bilingual research assistant (RA) conducted all study assessments and was blinded to treatment condition throughout the trial. The clinical records were maintained separately, and the RA was not included in clinical supervision meetings. Additionally, the interventionists and participants did not reveal their intervention assignment to the RA.

The interventionists were 2 master’s level clinicians with at least 2 years of experience working in the area of smoking cessation with Latinos. While the research assistants and study coordinator were blinded to group assignments, the interventionists could not be blinded due to their role in delivering the behavioral intervention. A treatment manual was developed for each of the 3 treatment interventions. The treatment manuals were used for the training and supervision of the interventionists. The Interventionist training process included sessions involving reading and reviewing the treatment manual, mock sessions with audio recording and review, and ongoing weekly supervision with the PI/clinical supervisor. Interventionists used printed forms with prompts during sessions. The interventionists were trained and supervised by a licensed practicing psychologist in the State of Texas with experience in conducting and supervising smoking cessation clinical trials with Latinos.

Participants were compensated for attending study visits with retail gift cards ranging from $50 to $100 based on the visit schedule with incremental increases. The protocol was approved by the Institutional Review Board at the University of Texas MD Anderson Cancer Center.

#### Health education control condition

The control condition consisted of a Health Education intervention and covered three separate health topics: (a) smoking and health, (b) nutrition/exercise, and (c) sleep hygiene. These sessions were primarily didactic and consisted of health education, followed by discussions and questions about the material and how it might relate to smoking. Participants also viewed selected videos related to the session’s topic, which were available in both English and Spanish. The investigative team had previously conducted health education control interventions [42, 43], which served as a model from which to replicate.

#### Culturally-tailored smoking cessation treatment

The CTSC intervention was developed based on Kreuter and colleagues’ [44] *Five Strategies Model of Cultural Tailoring* of health interventions which includes efforts to 1) design treatment materials (brochures and handouts) in a culturally-relevant manner with imagery that depicts Latino smokers; 2) emphasize the presentation of data that is directly related to Latinos (instead of the general population of smokers); 3) assure that materials and session content is delivered using language that is appropriate for Latinos in our catchment area; 4) directly draw upon the experience of members of the targeted group through the hiring of counselors that are Latino or have a history of working in the Latino community; 5) integrate health issues within the broader socio and cultural context including Latino cultural practices and values [45]. All components of the CTSC were linguistically appropriate (either in English or Spanish) based on participant preference. Several elements of the cultural tailoring replicated our previous work [27, 42, 43]. The first session was initiated with informal conversations about family and cultural background in order to enhance rapport and emphasize the Latino values of *respeto* (respect), *personalismo* (formal friendliness), and *familismo* (family values) [45]. These values have been consistently applied to behavioral health interventions and identified as salient among Latinos living in the US [45]. The counselor provided participants with an empirically validated smoking cessation self-help booklet for Latinos that consisted of linguistically accessible text and photographs about smoking, quitting, relapse prevention strategies, and personal testimonials [46].

Additionally, the importance of social support during a quit attempt was discussed, and the counselor worked closely with the participant to identify ways to enhance social support during a quit attempt, particularly through family support. Lastly, the counselor integrated pan- Latino values identified by G. Marin & B.V. Marin [45] into all aspects of the intervention including; (a) the association between Latino masculine ideals (*machismo*) and the use of pharmacotherapy; (b) the influence of *familismo* on smoking or quitting (responsibility to family, including setting examples for children); (c) the tendency towards *fuerza de voluntad* (willpower) as a primary means for achieving cessation; and (d) the role of *fatalismo* (a fatalistic outlook) regarding health and wellness. Such themes have also been recognized in the health literature to be particularly salient among Latino populations [47, 48].

#### Culturally-tailored smoking cessation treatment + adherence enhancement

The CT+AE intervention consisted of the aforementioned CTSC intervention with additional content related to NRT adherence. Specifically, the counselor facilitated didactic discussions to address misconceptions associated with NRT use and quitting, such as the health effects of the nicotine patch use, that have been identified as prevalent among Latinos [34]. The counselor also discussed the use of smoking cessation medication as it relates to several Latino cultural values [45] including health related fatalism and gender roles (*machismo* or *marianismo*). In previous studies [27], participants noted a high degree of shame associated with their smoking as well as unsuccessful attempts at quitting and the use of NRT. The Adherence Enhancement content also aimed to normalize the ambivalence often experienced with regard to quitting and the use of nicotine products as an aid for cessation in order to reduce shame and offer alternative modes of reframing their affective experience. Lastly, specific strategies for enhancing medication adherence were also discussed, including techniques for self-reminding and self-monitoring.

### Measures

#### Demographics

Participants completed a questionnaire that collected basic demographic information and asked about tobacco history; including the number of lifetime quit attempts and cigarettes per day (CPD).

#### Nicotine dependence

The 6-item Fagerström Test of Nicotine Dependence (FTND) was used to assess participants’ tobacco dependency [49, 50]. The FTND is a widely used measure which can be used to derive nicotine dependency level groupings: Low dependency (score 1–2), Low to Moderate dependency (score 3–4), Moderate dependency (score 5–7), and High dependency (score 8+).

#### Tobacco and NRT patch use

During each study visit, self-reported tobacco use was assessed using the Timeline Followback (TLFB), a calendar-based interview that asks participants to recall the frequency of substance use [51]. The TLFB has been shown to be a reliable measure of daily health activities, including substance use [52]. In the current study, participants were asked to report both tobacco use and the use of the nicotine patches. As a summary measure, we calculated descriptive statistics for the number of days of patch use, which served as our primary measure of NRT adherence. The smoking cessation literature has not established a level of NRT patch use that is associated with optimal smoking cessation rates [53–56]. However, previous studies have found that as NRT patch use/adherence increases, cessation rates improve [53]. In the current study, a level of ≥80% was applied as the clinically meaningful target of treatment.

#### Intervention acceptability

After completing each session, participants anonymously rated their level of satisfaction with specific session content and their overall satisfaction with the counseling session. The satisfaction survey used a 5-point Likert scale (from 1 = “Extremely Unsatisfied” to 5 = “Extremely Satisfied”). At the final session (Week 6 Visit), participants were asked to rate their level of satisfaction with treatment overall. Mean scores for each session were derived and used as a proxy measure of overall intervention acceptability, along with counseling session attendance.

#### Smoking status

Participants’ smoking was also assessed at each study visit using the *Society for Research on Nicotine and Tobacco Abstinence Status Questionnaire (SRNT)*, which captured self-reported 7-day point prevalence smoking verified with expired carbon monoxide (CO) testing using a Bedfont Micro+ Smokerlyzer [57]. A cutoff of 4 parts per million or greater was used to indicate a positive smoking result [58, 59]. This more conservative cutoff was selected based on previous studies indicating the need for more sensitive thresholds to account for “light” smokers [60] which were included in this study. Biochemically verified 7-day point prevalence smoking abstinence was selected as the primary measure of abstinence due to the fact that it is the most widely used variable in the smoking literature and would allow for the greatest level of comparability of findings. An intention-to-treat approach was applied with missed follow-up smoking assessments considered to be continued smoking.

### Analytic plan

The central aim of the study was to evaluate the feasibility and acceptability of our study procedures. As such, formal sample size calculations and estimations were not performed [61, 62]. The investigative team determined that a sample size of ≥8 per intervention arm was sufficient for obtaining feasibility data on recruitment, screening, treatment, treatment satisfaction and follow-up assessments procedures [61, 62]. Results are presented descriptively. All analyses were conducted using IBM SPSS Version 25 [63].

## Results

### Participant characteristics

Participant characteristics are summarized and presented in Table 1. The average age of the sample was 44.1 years (*SD* = 11.53. 60% of participants were male (*n* = 15) and 40% were female (*n* = 10). Most participants (*n* = 19; 76%) preferred speaking in Spanish and elected to complete their assessments and counseling sessions in Spanish. In terms of ethnic/cultural heritage, 16 participants (64%) identified as Mexican or Mexican American, 2 participants (8%) identified as Honduran, 2 participants (8%) identified as El Salvadorian, and the following nationalities were each represented by 1 participant (4%)–Peruvian, Cuban, Argentinian, Colombian, and Hispanic American (USA).

**Table 1:** Participant characteristics by intervention group.

	Intervention Group		
	HE (*n* = 9)	CTSC (*n* = 8)	CTSC+AE (*n* = 8)
**Age *M* (SD)**	45.4 (11.0)	46.0 (10.94)	40.7 (13.2)
**Female *n* (%)**	4 (44.4)	3 (37.5)	3 (37.5)
**Spouse/Partner *n* (%)**	7 (77.7)	4 (50.0)	7 (87.5)
**Employed *n* (%)**	6 (66.7)	6 (75.0)	6 (75.0)
**Years of Education, *M* (SD)**	12.7 (2.33)	11.3 (2.82)	11.2 (5.06)
**Baseline CPD, *M* (SD)**	13.2 (6.14)	15.7 (6.50)	11.5 (6.39)
**FTND Score**	3.8 (2.52)	5 (3.21)	2.87 (2.69)
**Lifetime Quit Attempts *M* (SD)**	3.33 (3.42)	2.12 (2.99)	4 (2.82)

Note: Nicotine replacement therapy (NRT). Health Education (HE); Culturally Tailored Smoking Cessation (CTSC); Adherence Enhancement (AE); cigarettes per day (CPD); Fagerström Test of Nicotine Dependence (FTND).

On average, participants smoked 13.4 cigarettes per day (*SD* = 6.33) and reported a mean of 3.1 (*SD* = 3.07) lifetime smoking quit attempts. The mean FTND score in the sample was 3.87 (*SD* = 2.69), which corresponded to the “*Low to Moderate*” range of dependence. FTND score groupings were distributed as follows: 52% low dependence (*n* = 13), 12% moderate dependence (*n* = 3), and 32% high dependence (*n* = 8).

### Intervention acceptability and feasibility

Retention rates for counseling sessions and follow-up assessments visits were strong overall. Across all treatment groups, 88% (*n* = 22) of participants returned for the Week 2 visit, and 84% (*n* = 21) returned for the Week 6 visit. The CTSC+AE group had a 75% (*n* = 6) of participants return for both the Week 2 and Week 6 Visits. Within the CTSC treatment group, return rates for the Week 2 and Week 6 visit were 87.5% (*n* = 7) and 100% (*n* = 8), respectively. All HE group participants (*n* = 9) returned for the Week 2 Visit and 77.8% (*n* = 7) attended the Week 6Visit. Follow-up assessment visit retention rates were also high, with 80% (*n* = 20) of participants returning for the 3-month follow-up and 76% (*n* = 19) returning for the 6-month follow-up.

Session satisfaction surveys scores were high in all three treatment groups for all three sessions assessed (See Table 2). Scores ranged from 4.40 to 4.83, corresponding to the response scale range of “Satisfied (4)” to “Extremely Satisfied (5)”. Furthermore, participants in all three groups rated their overall satisfaction with treatment highly (*M* = 4.80, SD = .402) with a mean corresponding to the “Extremely Satisfied” range.

**Table 2:** NRT patch use and session satisfaction survey means by treatment group.

	Intervention Group		
	HE (*n* = 9)	CTSC (*n* = 8)	CTSC+AE(*n* = 8)
**Days of NRT Patch use *M* (SD)**	64.6 (17.70)	68.6 (13.66)	81.3 (3.32)
**Overall Tx Satisfaction *M* (SD)**	4.87 (.35)	4.71 (.48)	4.83 (.40)
**Baseline Visit Satisfaction *M* (SD)**	4.66 (.40)	4.66 (.48)	4.40 (.96)
**Week 2 Visit Satisfaction *M* (SD)**	4.80 (.39)	4.71 (.39)	4.65 (.29)
**Week 6 Visit Satisfaction *M* (SD)**	4.83 (.32)	4.78 (.26)	4.66 (.43)

Note: Nicotine replacement therapy (NRT). Health Education (HE); Culturally Tailored Smoking Cessation (CTSC); Adherence Enhancement (AE); Treatment (Tx).

### NRT patch adherence

Overall, nicotine patch adherence was favorable; the full sample mean for the number of days of patch use (*M* = 70.5; *SD* = 15.4) was well above the NRT duration known to have a clinically meaningful effect [56]. As hypothesized, the CT+AE intervention group was found to have a greater number of days of patch use (*M* = 81.3; *SD* = 3.32) than both the CTSC condition (*M* = 68.6; *SD* = 13.66) and the HE condition (*M* = 64; *SD* = 17.70).

### Smoking abstinence

Smoking abstinence was assessed using 7-day point prevalence with expired carbon monoxide confirmation. At the conclusion of treatment, 28% (*n* = 9) of participants were abstinent, which consisted of 25% (*n* = 2) of the CT+AE group, 37.5% (*n* = 3) of the CTSC group, and 22.2% (*n* = 2) of the HE group (see Table 3). At the 3-Month Follow-Up Visit, a total of 10 participants were smoking abstinent; this consisted of 50% (*n* = 4) of the CT+AE group, 25% (*n* = 2) of the CTSC group, and 44.4% (*n* = 4) of the HE group. At the 6-Month Follow-Up visit, a total of 9 participants were smoking abstinent; this consisted of 37.5% (*n* = 3) of the CT+AE group, 25% (*n* = 2) of the CT group, and 44.4% (*n* = 4) of the HE group.

**Table 3:** Rates of biochemically verified 7-day point prevalence smoking abstinence by intervention group (*intention-to-treat*; N = 25).

	Intervention Group		
	HE (*n* = 9)	CTSC (*n* = 8)	CTSC+AE (*n* = 8)
**3 Month Follow-up n (%)**	4 (44.4)	2 (25.0)	4 (50.0)
**6 Month Follow-up n (%)**	4 (44.4)	2 (25.0)	3 (37.5)

Note: Nicotine replacement therapy (NRT); Health Education (HE); Culturally Tailored Smoking Cessation (CTSC); Adherence Enhancement (AE).

## Discussion

The current study represents the first known investigation using a three-group randomized control design and testing a culturally-tailored smoking cessation intervention for US Latinos that aimed to enhance adherence to NRT. While Latino smokers are known to have relatively low cessation rates and difficulty adhering to pharmacotherapy, findings from the current study demonstrate the preliminary acceptability and feasibility of our treatment interventions.

Participants rated counseling sessions favorably and attended sessions at a relatively high rate. Moreover, there were no serious adverse events associated with the trial. The HE, CTSC and CT+AE interventions were implemented effectively within a hospital-based clinic without any protocol deviations or significant problems pertaining to counselor training, supervision, or study oversight. As hypothesized, the CT+AE group had a higher level of NRT adherence. Although preliminary, this finding suggests that the targeted adherence strategies included in CT+AE may have contributed to the higher levels of NRT adherence. The CT+AE intervention was developed based on previous studies and theoretical frameworks involving Latino/Hispanic culture and health [44–48, 27, 28]which may have contributed to the findings. Nevertheless, the current findings are preliminary, and there is a strong need to test the intervention with larger and more diverse samples. Future studies should also explore the underlying mechanisms of action for the various content areas of the CT+AE intervention to enable ongoing adaptation and refinement.

Findings from the current study are unique in that all three treatment groups achieved relatively high rates of smoking abstinence at follow-ups (both 3- and 6-month). In fact, the biochemically-verified 7-day point prevalence abstinence rates among all three groups was equal to or greater than rates found in studies with the general population testing NRT, NRT + counseling, Buproprion, and Varenicline [64–66]. Moreover, in Webb and colleagues’ [17] review of smoking cessation trials with Latinos, the rates of biochemically-verified smoking abstinence at follow-up ranged from 17% to 22.5% among RCTs. If abstinence rates of the three groups in this trial were replicated in a larger and appropriately powered trial, the abstinence rate (>22%) would be comparable to those found in studies with the general population and Latinos.

Unexpectedly, our HE control group had an unusually high rate of smoking abstinence at follow-up as compared to the two culturally-tailored interventions. The design of the HE condition was intentionally time- and dosage-matched. Additionally, NRT was included as a background treatment in all three groups in order to accomplish one of the central aims of the study—test a smoking cessation intervention that targets pharmacotherapy adherence. As a result of these study design characteristics, the resulting HE control condition was highly “active” and a treatment effect was expected. The fact that the HE group ultimately showed the highest abstinence rate at 6-month follow-up, however, was unanticipated. Although there is insufficient data to make conclusions regarding possible causal factors associated with this finding, two potential contributing factors were considered. First, in previous studies of Latino smokers, less intensive interventions [24], control group interventions [67], and self-help interventions [20] have been shown to have statistically equivalent and comparable rates of smoking abstinence at follow-up compared to the experimental conditions Therefore, we considered the possibility that when treatment includes NRT, less-intensive counseling interventions may be just as effective for Latino smokers as more intensive counseling interventions. Secondly, it should be noted that participants in the HE condition received an intervention that was available in Spanish and delivered by Latina counselors who had experience working with Latino smokers. Possibly, these two elements alone, which correspond to domains of the *Five Strategies Model of Cultural Tailoring* Model [44], resulted in a culturally-tailored intervention capable of yielding outcomes of equal benefit to those seen in the other two treatment groups. Further research is needed in order to fully explore such potential contributing factors.

There were some notable baseline and treatment characteristic differences between groups that may have contributed to the HE group’s unexpected success. On average, and as compared to the CTSC and CT+AE groups, the HE group had a greater proportion of females (+6%), a mean education level approximately 1.5 years greater, and the highest rates of attendance for the second counseling session and both follow-up assessments. Given the pilot nature of the current study, it was not feasible to test whether these distinguishing characteristics may have had a moderating effect on the study outcomes. Yet, this finding may serve to inform future research.

Findings also showed a decrease in abstinence rates between the 3- and 6-month follow-up among the CT+AE group. Maintaining initial treatment effects beyond early follow-up is a well-documented challenge in the smoking cessation literature [17, 65, 66, 68]. Given the developmental nature of the current study, this finding serves as a rationale for including strategies to address the problem of sustaining initial treatment effects through to long-term follow-up in our CT+AE intervention. Previous studies have tested smoking relapse prevention strategies, including the extension of pharmacotherapy [68] and adding follow-up telephone booster sessions [69,70]. In future studies, the addition of booster sessions and extended NRT can be easily incorporated into the CT+AE intervention, and the impact on long-term cessation outcomes can be evaluated.

Several limitations should be carefully considered when interpreting findings of the current study. Foremost, as a feasibility pilot randomized trial, the small sample size limited our ability to conclusively test hypotheses and investigate underlying mechanisms relating to our findings. While the study demonstrated feasibility and acceptability of the interventions and led to preliminary findings on their effects, a larger trial is needed to establish the efficacy of these interventions. Secondly, the sample was limited to adult Latino smokers residing in our catchment area, of which 64% were of Mexican origin. As such, the results may not generalize to the general population of Latino smokers in the U.S., which includes a variety of ethnic, regional and national heritages. Despite this limitation, the sample was comparable with the most recent U.S. Census estimates of the various ethnic subgroups of the adult Latinos population [71]. Therefore, these findings are considered to have relatively high generalizability to the general Latino population in the United States. Third, due to the nature of the interventions tested in this trial (behavioral interventions) the study interventionists could not be blinded to treatment condition as were the RA’s and study coordinator. The lack of control of this variable may have impacted the delivery of the interventions and results. Fourth, the current study involved self-reported measurement of NRT adherence. This approach may have under- or over-represented participant adherence. A more rigorous approach for capturing adherence data such as medication event monitoring systems could have validated self-reports. Furthermore, future research should address potential confounding variables that this feasibility pilot randomized trial could not address or examine, such as motivation for treatment, the effect of visits that involve CO testing (monitoring) and participation incentives, and whether findings would be similar with a waitlist control group or Latinos living outside of our catchment area. Nevertheless, our findings are comparable to previous trials of smoking cessation behavioral interventions that typically include CO testing and participant incentives [66].

## Conclusions

Despite these limitations, the current study provides preliminary evidence for the feasibility, acceptability, and efficacy of a brief, culturally-tailored, and adherence-enhancing smoking cessation intervention for Latino smokers. This pilot RCT represents the first investigation specifically targeting Latino smokers using a culturally-tailored intervention with a focus on adherence enhancement. Findings from this study were promising, and the rates of smoking abstinence across all three treatment groups were favorable and worthy of further research. Considering the prevalence of smoking among Latino adults [2] and the extent to which smoking impacts health [4], the current study represents a significant contribution to the health literature. Previous smoking cessation studies with Latinos have struggled to attain even modest levels of smoking cessation at follow-up [17]. Future studies may draw upon the current findings and augment existing smoking cessation treatments with both culturally-tailored content as well as content aimed at enhancing adherence to smoking cessation pharmacotherapies among Latinos.

## Supporting information

S1 File IMPACT protocol.pdf. This is the study protocol file.
(PDF)

S2 File DATA.xpt. This is the study data file.
(XPT)

S3 File CONSORT 2010 Checklist_Revision 11.1.18doc(1).docx. This is the CONSORT Checklist file.
(DOCX)
