# Supplementary material for: A pilot randomized trial examining the feasibility and acceptability of a culturally tailored and adherence-enhancing intervention for Latino smokers in the U.S
Source: PLoS One. 2019 Jan 11;14(1):e0210323. doi: 10.1371/journal.pone.0210323 (PMC6329496; doi:10.1371/journal.pone.0210323)
Supplement: S1 File — This is the study protocol file. (PDF) [file pone.0210323.s001.pdf]

PDOL Working Copy

THE UNIVERSITY OF TEXAS
MD Anderson ~~Cancer~~ Center

Protocol Page

Project Impact 2: A Culturally Tailored Smoking Cessation Intervention for Latino Smokers
2015-0585

Core Protocol Information

**Short Title**	Project Impact 2
**Study Chair:**	Marcel A. de Dios
**Additional Contact:**	Loren G. Bryant<br>Sarah D. Childress
**Department:**	Health Disparities Research
**Phone:**	713-745-0296
**Unit:**	1440
**Full Title:**	Project Impact 2: A Culturally Tailored Smoking Cessation Intervention for Latino Smokers
**Protocol Type:**	Standard Protocol
**Protocol Phase:**	Phase I/Phase II
**Version Status:**	Working copy
**Version:**	05

Which Committee will review this protocol?

(•) The Psychosocial Behavioral and Health Services Research Committee (PBHSRC)

Protocol Body

1.0 Objectives

The objective of this study is to conduct a pilot 3-group randomized clinical trial involving three behavioral health interventions for smoking cessation among Latino smokers. Participants will be randomized to one of three interventions: A 1) Health Education (HE) control condition, a 2) Culturally Tailored Smoking Cessation (CTSC) condition or a 3) CTSC + Adherence Enhancement (AE) condition. All three conditions will include three behavioral intervention sessions with a counselor at baseline, 2 weeks and 6 weeks. Participants in all three groups will also receive 12 weeks of Nicotine Replacement Therapy in the form of nicotine patches. We will assess cessation at follow-up which will occur at 3 and 6 month follow up. The specific aims of this project are:

Aim 1: Evaluate the feasibility, fidelity, and acceptability of the CTSC and CTSC+AE in Latino smokers. Our evaluation of these domains will seek to identify elements of the intervention that are successful or in need of improvement for a planned larger trial. To evaluate these domains we will assess: 1) Recruitment and retention of participants 2) Intervention adherence 3) Program satisfaction 4) The extent to which the intervention was delivered as planned (derived from the Fidelity Checklist [Appendix Z], described in the measures section).

Aim 2: To evaluate the preliminary efficacy of the CTSC and CTSC + AE, as compared to HE, on increasing biochemically verified 7-day point prevalence smoking abstinence rates at 3 and 6 month follow-up in Latino smokers.

Aim 3: To evaluate the efficacy of the CTSC+AE condition, as compared to the HE and CTSC groups, on increasing adherence to nicotine patch treatment (NRT adherence is defined as the proportion of days of NRT use over the number of patches made available).

Aim 4 (Exploratory): To examine potential mediators and moderators of the relationship between our primary outcomes (Aims 1-3) and our treatment groups. For this exploratory aim we will examine several variables that may influence treatment outcomes including gender, age, alcohol use, health related fatalism, sense of control and acculturation.

2.0 Background

Tobacco Use and Smoking Cessation among Latinos

Latinos are the largest and fastest growing ethnic group in the United States (US)[43]. They constitute 16% of the population and by the year 2030, it is estimated that 20% of individuals residing in the US, will be Latino.[44] Smoking has been identified as a significant and concerning public health problem among Latinos. In a recent national

survey of Latino health, an overall smoking prevalence rate of 16.9% was found with substantial disparities by gender and national origin.[1] For example, smoking among Mexican American males (23.4%), Cuban males (31.3%) and females (21.9%), as well as Puerto Ricans males (35%) and females (32.6%) exceeds the smoking prevalence rate of non-Latino Whites (19.4%). Three of the four leading causes of death among Latinos are associated with smoking (cancer, heart disease, & stroke).[2] Moreover, lung cancer is the foremost cause of cancer death for Latino men and the second leading cause for Latina women.[1,2]

Latino smokers experience smoking cessation treatment disparities due to a general lack of health care access,[3-6] under-utilization of existing services,[7] and poor treatment outcomes.[8-10] Various national surveys have determined that Latinos receive advice to quit smoking from their physician at lower rates than other ethnic/racial groups.[11-14] In multiethnic/racial treatment studies, Latinos have been shown to have lower smoking cessation abstinence rates as compared to Whites.[8-10] A relatively limited number of studies have tested smoking cessation interventions for Latino smokers.[15-18] Of the existing studies, only six utilized an RCT design that included a control group and biochemical measures of smoking abstinence at follow-up.[19-23] In Webb and colleagues (2010) meta-analysis that included five of these six studies[15], an odds ratio effect size (ESOR) of 1.54 (95% CF, 1.09-2.16) was calculated, indicating that the experimental treatments in the selected studies did have a significant effect on cessation rates. However, this effect was only found at the *end of treatment* and deteriorated by follow-up.[15] Furthermore, the extent to which the treatments in these studies were actually culturally tailored to Latinos is questionable and raised as an area in need of further research.

It has been over 7 years since the US Department of Health and Human Services published its updated *Clinical Practice Guideline for Treating Tobacco Use and Dependence* (2008)[45] which urged the advancement of research focusing on the development and testing of culturally adaptive smoking cessation for racial minority populations including Latinos. Since that time, there have only been three published RCTs of smoking cessation interventions for Latinos.[16-18] Of the three, two of the studies (both from our own group)[16-18] specifically sought to tailor an intervention to the unique cultural characteristics of Latinos. Considering the lack of research attention and the disparities in treatment among Latinos, smoking is likely to continue being a significant public health problem if further research is not conducted.

Adherence to Smoking Cessation Treatment among Latinos

Studies have sought to explore the unique characteristics of Latino smokers that may influence the success of smoking cessation treatment.[24,25] Levinson et al.,[25] demonstrated that Latinos are less likely to use pharmacotherapy treatments for smoking cessation than other ethnic/racial groups, even after controlling for factors such as health care access, income, physician advice to quit, health status and

smoking level. In a follow-up study Levinson et al.,[24] identified a number of potential barriers of Latinos with regard to pharmacotherapies including: concerns about side effects, fears of becoming dependent on medications, cultural inclinations towards quitting without chemical aid, a lack of knowledge about the effectiveness and use of medications, and misconceptions about the perceived risks of smoking.[24]

Nicotine replacement therapy (NRT), the most commonly used and available smoking cessation pharmacotherapy aid, has been found to increase the likelihood of quitting by up to two-fold.[46-48] However, studies have shown that when smokers are non-adherent to the full course of NRT there are lower follow-up abstinence rates.[26-28,49] Latinos are particularly wary of utilizing any pharmacological aids for smoking cessation[24]; therefore they are prone to NRT non-adherence.[24,25] In our prior work, Latinos were found to have 56.6% adherence to a 12-week NRT patch regimen which is a considerably lower treatment dose than the recommended minimum practice guideline.[45] The challenge of maintaining medication adherence among Latinos is not exclusive to NRT or smoking cessation. As compared to other ethnic/racial groups, Latinos have lower adherence rates to medications for chronic medical conditions[29] such as diabetes[50], hypertension[51,52], HIV/AIDS[53], and psychiatric conditions.[54]

Medication adherence poses a significant barrier to smoking cessation treatments for Latinos and the unique needs and concerns of Latino smokers who seek to quit requires the development of culturally tailored interventions. Previous studies seeking to develop Latino specific interventions have emphasized linguistic appropriateness, integrating values that are specific to Latinos, acculturation, and addressing specific gaps in knowledge or cultural traditions that may be barriers to treatment.[21,22,30-35,55] These cultural domains have yet to be fully integrated with strategies for increasing adherence to smoking cessation treatment. The current study seeks to test a culturally tailored smoking cessation intervention + an adherence enhancement add-on intervention among Latinos smokers.

3.0 Background Drug Information

The efficacy of the nicotine patch has been extensively studied and each of the published meta-analyses concluded that the patch increased rates of smoking cessation.[56-59] Nicotine patches will be purchased from an outside vendor and/or through the MDA Pharmacy. The patch used in this protocol is approved for over-the-counter sale and use. Due to the use of nicotine patches, taking part in this study can result in risks to an unborn or breastfeeding baby, and you should not become pregnant or breastfeed a baby while on this study.

4.0 Patient Eligibility

Participants & Recruitment: A total of 36 participants will be enrolled, 12 in each condition. We will recruit participants by posting flyers at locations in the greater Houston area that are known to be frequented by our target Latino population (ex: community centers, Hispanic food markets and grocery stores, salons, bus stations, clinics serving mainly Hispanics). We will also post ads in free Houston area Spanish language print newspapers (ex: La Subasta, Buena Suerte) as well as use radio ads heard throughout the greater Houston area. All ads and flyers will use the IRB approved advertisements. (See Appendix R).

Inclusion Criteria:

1) Adults at least 18 years of age

2) Current smoker (>5 cigarettes per day for the past 3 months)

3) Able to speak and read English or Spanish

4) Agree to participate in the study and be available for 6 weeks of treatment and 6 months of follow-up

5) Willing to set a quit date within 2 weeks of enrollment date

6) Identify as being of Latino heritage, ethnicity, or ancestry

Exclusion Criteria:

1) Individuals suffering from any unstable medical condition precluding the use of NRT (Identified using the Medical History Questionnaire given at baseline, See Appendix X)

2) Currently using smokeless tobacco, electronic nicotine delivery systems (ENDS), nicotine replacement therapy, or other smoking cessation treatment

3) Pregnant or nursing

4) Suffering from a severe psychiatric disorder (assessed using self-reported history of psychiatric diagnosis during the phone screening) that would interfere with participation

5) Diagnosis of substance dependence other than nicotine (screened using DSM IV TR criteria)

6) Individuals that do not have access to a working telephone

5.0 Pretreatment evaluation

Recruitment: Participants will be recruited through the recruitment strategies previously discussed in *Section 4.0: Patient Eligibility*. Potential participants will be screened over-the-phone by a research assistant using a checklist of the study inclusion and exclusion criteria (See Appendix C). Eligible and interested participants will be scheduled for a baseline visit, which will last approximately 2.5 hours at M.D. Anderson. Potential participants will be informed that the visit will entail a series of questionnaires and a one-on-one session with a counselor regarding their smoking and quitting. Participants will be informed that the entire visit will last approximately 2.5 hours and they will be compensated with a $50 gift certificate. The day prior to their visit to M.D. Anderson, participants will receive a reminder call from a research assistant. The reminder call or voicemail message will not divulge any information regarding their participation in the study.

Baseline Visit: Study personnel will provide a detailed description of the study, answer questions, obtain written informed consent, and finalize eligibility. Participants will complete a series of questionnaires at baseline, and a trained research assistant will collect a sample of expired carbon monoxide using a Bedfont Micro+ Smokerlyzer®. Participants who are able to become pregnant will be required to take a routine hCG urine sample pregnancy test administered by the research assistant to rule out potential pregnancy.

Measures The assessment battery will be available in both English and Spanish languages. Based on our previous work with the proposed instruments, we estimate the battery of measures will take approximately 1.5 hours to complete at baseline and during the 3 and 6-month visits.

Measures	Approximate time to complete (minutes)	Total for session
**Baseline Visit**		
Pregnancy Test (Females only)	5	
Demographics	20	
Medical History	5	
Tobacco History	10	
Fagerstrom (FTND)	5	
SRNT Abstinence Status	15	
Alcohol Quantity & Frequency	5	
Sense of Control	5	
Religious Health Fatalism	5	
Bicultural Involvement (BIS)	10	
Smoking Status	2	**87 minutes**

**Counseling Sessions & Follow-Up**		
Adverse Events Monitoring (Completed with RA)	10	
Adherence-TLFB (Completed with RA)	20	
Smoking Status	2	
Fagerstrom (FTND)	5	
Alcohol Quantity & Frequency	5	
Sense of Control	5	
Religious Health Fatalism	5	
Bicultural Involvement (BIS)	10	
Satisfaction Surveys (counseling sessions only)	5	**67 minutes**

Demographics Questionnaire (Appendix D) administered at baseline will collect data on age, race, ethnicity, education, income, preferred language, and generations in the U.S. This questionnaire has been used extensively by our team.

Medical History (Appendix X) Participants will be asked to provide a detailed medical history including heart disease, asthma/lung disease, high blood pressure, diabetes, high cholesterol, thyroid problems, and kidney disease. Participants will also be asked to provide a detailed list of medications and their dosage that they take daily.

Tobacco History (Appendix E) collects information regarding onset of regular smoking, previous quit attempts, abstinence history, smoking rate, other household smokers, proportion of friends/family who smoke, and other tobacco use. This questionnaire has been used extensively by our team.

The *Fagerstrom Test for Nicotine Dependence* (FTND) (Appendix F) is a widely used measure of nicotine dependence. The Heaviness of Use Index is composed from a portion of the items on the larger measure.[61,62] Six questions from the Fagerstrom questionnaire will be used in this protocol.

Society for Research on Nicotine and Tobacco (SRNT) Abstinence Status Questionnaire (pre- and post-versions) (Appendix G) surveys tobacco use by the participant and persons within the participant's household and social surroundings.[63]

The *Alcohol Quantity and Frequency Measure* (Appendix H) assesses quantity and frequency of alcohol consumption and is adapted from Cahalan (1973).[64]

Sense of Control (Appendix I) balances claiming versus denying control statements for good and for bad events, cancels the agreement bias that commonly exists when using

Likert-scaled items, and improves the validity of the sense of control measure.[65]

Religious Health Fatalism Questionnaire (RHFQ) (Appendix J) Religious Health Fatalism is defined as “the belief that health outcomes are inevitable and/or determined by God”, and assess the association between Religious Health Fatalism and health behaviors among individuals.[66]

Bicultural Involvement Scale (BIS) (Appendix K) is a 24-item self-report measure used to assess cultural orientation. The measure generates scores for individuals' independent association with each of the two cultures, their degree of biculturalism, and a rating of their total cultural involvement. Originally developed for use with Hispanic and American individuals, the measure has also been modified for use with individuals from other cultures, including Asia and Australia. Adapted from the acculturation scale, the bicultural involvement questionnaire was developed by José Szapocznik, William M. Kurtines, and Tatjana Fernandez in 1980 as a multidimensional tool for measuring cultural orientation. Unlike the majority of acculturation measures that preceded it, the BIQ does not assume that involvement in one culture necessitates exclusion of association with another.[67]

Smoking Status will be assessed at each study visit. Self-reported abstinence will be verified using expired carbon monoxide testing using a Bedfont Micro+ Smokerlyzer® at the 2 and 6-week sessions and 3 and 6-month follow-up assessments.

Randomization: After the Baseline Visit, participants will be randomly assigned to CTSC, HE, or CTSC+AE using a standard urn randomization technique (1:1:1) stratified by gender.

6.0 Treatment Plan

The proposed pilot study is a 3-group RCT of 36 Latino smokers with all participants receiving 12-weeks of nicotine patch treatment and self-help materials. Participants randomized to our experimental conditions (CTSC and CTSC+AE) will receive 3 individual counseling sessions incorporating Latino cultural tailoring (described below). The CTSC+AE group will receive counseling session content that specific emphasizes techniques and strategies for improving NRT adherence as well as discussions relating to the emerging themes and issues garnered from the qualitative portion of this study (Project Impact 2, Protocol #2015-0585) The HE control condition will also receive 3 sessions with a counselor focused on general health education and smoking. Treatment manuals for HE, CTSC, and CTSC+AE will be used to ensure standardization of treatment delivery as well as to provide a basis for evaluating therapist competence and adherence. All participants will attend 2 follow-up assessment visits occurring at 3 and 6 months post-treatment. Randomization: Participants will be randomized into one of three treatment conditions in a 1:1:1 distribution (CTSC, CTSC+AE or HE). Compensation: Participants will be compensated for all study visits with gift certificates. The compensation schedule will be as follows: Baseline assessment-$50; Session 2-$40; Session 3-$60; 3-month follow-up $75; and $100 for the 6 month follow-up. Participants that drive to the medical center will be reimbursed for parking; those using public transportation will be given public transit passes.

Treatment Components Included in Both Conditions: Participants in all three arms of the study will receive Nicotine Replacement Therapy (NRT), which is the most commonly used and available smoking cessation pharmacotherapy. The use of NRT during a quit attempt is associated with a 50-70% increase in cessation rates.[37] Thus, NRT is a well-established and empirically supported first-line treatment for individuals seeking pharmacotherapy for smoking cessation.[36] Furthermore, when NRT is coupled with a behavioral intervention such as counseling, cessation rates substantially increase by up to 16%.[37] Therefore, the combination of a behavioral intervention with NRT is widely considered an optimum level of treatment. All participants (CTSC, CTSC+AE and HE conditions) will receive 12 weeks of NRT patch treatment - Nicoderm CQ (GlaxoSmithKline, Philadelphia, PA). Participants will be advised to use the patch according to the standard tapering schedule: 4 weeks of 21 mg, 4 weeks of 14mg, and 4 weeks of 7mg patches. Patch dispensation will occur at study visits. Participants receive only the number of patches necessary to last until the next study visit plus several extra patches should a patch fall off, become torn, etc., or should the visit be delayed. Based on our previous research, providing subjects with only enough patches to last until the subsequent visit improves compliance. A reduction in dosage or cessation of the patch regimen will be implemented for any participant who shows signs of being on too high of a dose. This is typically necessary for very few participants because blood nicotine levels are usually much lower on the patch than while smoking.[68] Self-Help Materials: Self-help materials will consist of the consumer products

developed for the *Treating Tobacco Use and Dependence Clinical Practice Guideline*.[38]

During the first session and irrespective of treatment condition, the interventionist will work with the participant to set a quit date within two weeks (participant's willingness to set a quit date within 2 weeks is an inclusion criteria for the study). The process of setting a quit date will follow the *Treating Tobacco Use and Dependence Clinical Practice Guidelines*[38] and the interventionist will engage in a discussion about the ideal date and the consideration of various scheduling factors (e.g., a weekend date versus weekday).

<u>Health Education Control Condition:</u> We will use a Health Education intervention as our control condition. The duration of the three health education sessions and their timing (after the assessment at baseline, 2 and 6 weeks) will be identical to the experimental conditions. The 3 sessions will cover: 1) smoking and health, 2) nutrition/exercise (Appendices U/W) and 3) sleep hygiene (Appendix V). These sessions will primarily be didactic and consist of health education, followed by discussions and questions about the material and how it might relate to smoking. Our investigative team has conducted such health education interventions in previous studies.[39,40] We will replicate this work and develop handouts and illustrations for the HE sessions. See Appendix S for HE manual.

<u>Culturally Tailored Smoking Cessation Treatment:</u> The CTSC intervention has been developed based on Kreuter and colleagues' (2003)[69] *Five Strategies Model of Cultural Tailoring* of health interventions. The five strategies are: 1) Peripheral Strategies– design materials and programs so as to make them appear culturally relevant; For example, the use of brochures depicting images of Latino smokers instead of non-culturally specific imagery. 2) Evidential Strategies – the presentation of evidence to participants that is directly related to their respective group. For example, rather than simply stating that smoking causes cancer, a counselor may say "16.9% of Latinos smoke and lung cancer is the leading cause of cancer death among Latino men." 3) Linguistic – assuring that materials and programs are in the language of your select group. This is self-explanatory. 4) Constituent-Involving Strategies – approaches that aim to directly draw upon the experience of members of the targeted group. For example, hiring counselors that are Latino or have a history of working in the Latino community. Lastly, 5) Sociocultural strategies attempt to integrate health issues within the broader socio and cultural context. For example, integrating elements of the target group's cultural values, beliefs, and behaviors during discussions.[69] (See Appendix T for CTSC manual)

All components of the CTSC will be linguistically appropriate (either in English or Spanish) and delivered by counselors who are Latino and have clinical experience in smoking cessation as well as in working with Latinos in Houston. Other elements of our cultural tailoring will replicate our previous work[16,39,40] and the first session will be initiated with informal conversations about family and cultural background in order to enhance rapport and emphasize the Latino values of *respeto, personalismo*, and *familismo.*[41] The

counselor will provide participants with an empirically validated smoking cessation self-help booklet for Latinos – "*No Lo Deje Para Mañana, Deje De Fumar Hoy – Una Guía Para Dejar De Fumar*" (NIH Publication No. 02-3001) (Appendix O).[34] This manual ("Guía") consists of 36 full color pages of simple text and photographs that includes information about smoking, quitting, relapse prevention strategies, and personal testimonials. The content of the Guía was specifically developed to be culturally sensitive to Latinos and was found to be acceptable during the focus group phase of *Project Aurora*. The Guía has also been shown to improve verified quit rates among Latinos at one-year follow-up.[34] In addition, the importance of social support during a quit attempt will be discussed and the counselor will work closely with the participant to identify ways to enhance social support particularly through family, during the quit attempt. Lastly, the counselor will integrate pan-Latino values identified by Marin & Marin (1991)[41], into all aspects of the intervention. For example *machismo* (association between masculine ideals and the use of pharmacotherapy, smoking and *familismo* (responsibility to family, including setting examples for children), and the de-emphasis of *fuerza de voluntad* (willpower) as the primary means for achieving cessation, as well as the de-emphasis of *fatalismo* (fatalistic outlook) of health and wellness have all been recognized in the literature and will be integrated into session content.

<u>Culturally Tailored Smoking Cessation Treatment + Adherence Enhancement</u>

The CTSC+AE intervention will consist of the above described CTSC intervention with additional content and discussions related to NRT adherence. Specifically, the counselor will lead didactic discussions regarding the prevalent misconceptions associated with NRT use and quitting that have been identified among Latinos.[24] For example, the counselor will discuss the common misconceptions regarding the health effects of the nicotine patch use. In addition to addressing misconceptions, the counselor will also explore the topic of smoking cessation medication use as it relates to several Latino cultural values[41], including health related fatalism, gender roles (machismo or marianismo), and health related bravado.[42] Throughout the AE content the counselor will also aim to normalize the ambivalence that is often experienced with regard to quitting and the use of a nicotine products as an aid for cessation. In our preliminary work, participants noted a high degree of shame associated with their smoking as well as unsuccessful attempts at quitting and the use of NRT. The normalization of these areas will help to alleviate shame and offer alternative modes of reframing their affective experience. Lastly, specific strategies for enhancing medication adherence will also be discussed including techniques for self-reminding and monitoring.)See Appendix T for CTSC+AE manual).

The interventionist will be a master's level clinician with at least 2 years of clinical experience. Dr. Marcel de Dios (PI) will train and supervise the interventionist. Prior to the initiation of the study, Dr. de Dios will conduct one-on-one training sessions with the interventionist which will cover the execution of the protocol specifically the treatment interventions. Training sessions will include role plays and feedback. Once the study is initiated, Dr. de Dios will meet with the interventionist on a weekly basis to discuss any

emergent issues that need to be addressed through further training. Dr. de Dios will review all session audios and use a checklist based on the treatment manual to maintain treatment fidelity and address any therapist "drift". Dr. de Dios is a licensed and practicing psychologist in the State of Texas and has had experience in conducting and supervising behavioral interventions in the context of smoking cessation trials.

7.0 Evaluation During Study

These measurements will be taken during each counseling session visits at week 2 and 6, and at the 3 and 6-month follow-up visits.

Measures: As noted previously, the assessment battery will be available in both English and Spanish languages. Based on our previous work with the proposed instruments, we estimate the battery of measures will take approximately 1.5 hours to complete at baseline and during the 3 and 6-month visits.

Adverse Events Monitoring Participants will be closely monitored for any adverse side effects. Each in person visit that participants attend will include comprehensive assessment of adverse events, side effects and medication tolerability. Any reported adverse events will be kept in a log by the counselor or research assistants and reported to the IRB as necessary (Appendix L). Throughout the entire study, participants will be reminded that if they experience any intolerable or problematic side effects, they should immediately contact Dr. de Dios and discontinue using NRT. During the event that a participant has a mental health emergency during a study visit session, the present research assistant will follow the Mental Health Emergency Procedures instructions (Appendix P).

Adherence NRT adherence will be assessed using three methods: 1) The Timeline Follow-back (TLFB)[70] (Appendix M) interview at monthly visits. In our preliminary studies we successfully used the TLFB to collect data on medication adherence.[16] The TLFB is a calendar-based interview that asks participants to recall the frequency of substance use. The TLFB has been used extensively in assessing the use of a variety of substances and health behaviors. This interview procedure involves eliciting calendar guided recall and utilizing anchoring of dates to significant events. The TLFB is widely used in the field of substance use research and has been shown to be a reliable measure of daily activity associated with a variety of health behaviors. 2) Participants will be asked to keep an NRT calendar diary that will be collected at each study visit. 3) Participants will also be asked to bring in their boxes of patches and the clinical research assistant will conduct a count of used and unused patches. We are likely to encounter instances when our 3 adherence indicators are not consistent. In such cases, we will utilize the lowest adherence value obtained. The NRT adherence variable will be computed as the proportion of days of NRT use over the number of patches made available.

Satisfaction Survey of Session Elements (Appendix Y)This is an investigator developed

scale on a 5 point Likert scale to assess to the extent to which the participants were satisfied with various elements of the session content. The items reflect the content of the HE, CTSC, and CTSC-AE manuals.

Smoking Status will be assessed at each study visit. The *Society for Research on Nicotine and Tobacco (SRNT) Abstinence Status Questionnaire* (pre- and post-versions) will be used. It surveys tobacco use by the participant and persons within the participant's household and social surroundings.[63] Our outcome measure will be self-reported 7-day point prevalence smoking abstinence verified by expired carbon monoxide testing using a Bedfont Micro+ Smokerlyzer®. This variable will be assessed at the 2 and 6-week sessions and at the 3 and 6-month follow-up assessments.

The *Fagerstrom Test for Nicotine Dependence* (FTND) is a widely used measure of nicotine dependence. The Heaviness of Use Index is composed from a portion of the items on the larger measure.[61,62] Six questions from the Fagerstrom questionnaire will be used in this protocol.

The *Alcohol Quantity and Frequency Measure* assesses quantity and frequency of alcohol consumption and is adapted from Cahalan (1973).[64]

Sense of Control balances claiming versus denying control statements for good and for bad events, cancels the agreement bias that commonly exists when using Likert-scaled items, and improves the validity of the sense of control measure.[65]

Religious Health Fatalism Questionnaire (RHFQ) Religious Health Fatalism is defined as "the belief that health outcomes are inevitable and/or determined by God", and assess the association between Religious Health Fatalism and health behaviors among individuals.[66]

Bicultural Involvement Scale (BIS) is a 24-item self-report measure used to assess cultural orientation. The measure generates scores for individuals' independent association with each of the two cultures, their degree of biculturalism, and a rating of their total cultural involvement. Originally developed for use with Hispanic and American individuals, the measure has also been modified for use with individuals from other cultures, including Asia and Australia. Adapted from the acculturation scale, the bicultural involvement questionnaire was developed by José Szapocznik, William M. Kurtines, and Tatjana Fernandez in 1980 as a multidimensional tool for measuring cultural orientation. Unlike the majority of acculturation measures that preceded it, the BIS does not assume that involvement in one culture necessitates exclusion of association with another.[67]

Fidelity Checklist (Appendix Z) is an investigator developed checklist that mirrors the content of the HE, CTCS, and CTSC+AE treatment manual. This checklist will be used by the PI or his designee to review each session audio and document whether or not each element of the treatment manual was delivered by the interventionist. The Fidelity

Checklist will provide descriptive data on content delivery to aid in modifying our intervention in subsequent studies. It will also be used as a tool for clinical oversight and the ongoing monitoring of interventionist. As needed, the PI will provide further training to the interventionist if substantial intervention "drift" is identified.

Distress Plan

In addition to the treatment parts listed above, participant distress during assessment and treatment sessions will be actively monitored and addressed by all study personnel (research assistant, interventionist, and the PI). Dr. Marcel de Dios (PI) will train and supervise the research assistant (RA) and the interventionist prior to the initiation of the study. Training will not only include the execution of the protocol but also, handling participant distress and psychiatric emergencies. Training sessions will include role plays and feedback. Once the study is initiated, Dr. de Dios will meet with the RA and interventionist on a weekly basis to discuss any emergent issues that need to be addressed through further training.

If a participant experiences significant emotional distress during their study (both assessment and treatment visits) they will be referred to an appropriate mental health provider. If they report or present with any severe psychiatric symptoms such as suicidality an emergency protocol will be enacted which includes assessment for hospitalization (See Appendix P: Mental Health Emergency Procedures). Dr. de Dios is a licensed clinical psychologist in the States of Texas and Rhode Island and has experience in assessing, managing, and supervising staff on procedures for addressing psychiatric emergencies.

8.0 Evaluation of Toxicity

Not applicable.

9.0 Criteria for Response

Not applicable.

10.0 Criteria for Removal from the Study

Subjects who are excluded at the initial screening or withdraw from the study will be given self-help smoking cessation materials and will be referred to alternate smoking cessation programs in the area. Participants may be removed from the study at the PI's discretion for any of the following reasons:

1) The participant's behavior disrupts others or prohibits others from receiving and/or benefiting from the treatment.

2) The participant begins the use of smokeless tobacco, electronic nicotine delivery system (ENDS), or other nicotine replacement therapies (NRT) during the course of the study.

3) The participant becomes pregnant during the course of the study.

11.0 Statistical Considerations

Proposed Analyses: In this pilot study, we will summarize descriptive statistics to characterize our study sample and describe between group differences. Descriptive statistics will be tabulated for all study variables which includes demographics, measures of intervention acceptability, feasibility, fidelity, and smoking history, nicotine dependence, and other variables.

We also plan to conduct basic effect size estimates to derive preliminary findings based on pre/post design. With such a small sample size, we will focus on the substantive magnitude of observed between group differences. We will, however report Fischer's exact p-values and the nonparametric Kruskal-Wallis test for equality of rank-ordered population distributions when comparing treatment groups on categorical and continuous outcomes, respectively. Graphical methods will also be used to explore and describe the association between adherence to treatment and smoking in each of the intervention groups.

Aim 3 compares the three treatment groups (CTSC, CTSC+AE, & HE) on the mean percentage of days of NRT adherence during the 12 weeks of treatment. In our preliminary study of Latino smokers (Project Impact) we found that on average participants were adherent on 46.7% (standard deviation=36) of days during the 12 weeks of NRT treatment. Based on this finding, we estimate that our control condition (HE) will show a similar rate of NRT adherence (adherent on 56% of treatment days). We are guided primarily by our expectations that clinically significant improvements in outcomes will be achieved with >75% adherence and we base our estimate of the NRT adherence rate for our CTSC+AE condition on two studies reporting NRT adherence rates for interventions that sought to increase adherence but did not exclusively target adherence. The adherence rates of the experimental condition in these studies ranged from 88-93%. The current study is not only designed to test the feasibility of CTSC and CTSC+AE (Aim 1), but to evaluate the CTSC+AE intervention compared to HE and CTSC groups. It is not expected to be a definitive efficacy trial and the proposed sample will yield power to detect only large effects. We will have power to detect a conservative estimate of 80% adherence in our CTSC+AE condition. When we account for an attrition rate of 20%, a sample of 36 participants can detect a mean difference of 13% or greater between the groups using a 2-tailed alpha = .05. Due to the limited scope of this development project, this study will not be powered for formal hypothesis testing of group differences. Nevertheless, the proposed study will conduct analyses investigating distributional assumptions and co-variation among examined predictors which will inform the design of a future efficacy trial that will be fully powered. Lastly, we will explore potential mediators and moderators (AIM 4) of our primary outcomes and treatments using descriptive statistics, the chi2 test, and correlational analyses including the bivariate Pearson r. This approach is unable to confirm a significant mediational or moderation effect. It was selected due to our limited sample size. However, our aim is to identify potential trends that can be further tested in a larger and

subsequent study using standard mediational and moderation analytic approaches.

Aim 1: Evaluate the feasibility, fidelity, and acceptability of the CTSC and CTSC+AE in Latino smokers. Our evaluation of these domains will seek to identify elements of the intervention that are successful or in need of improvement for a planned larger trial. To evaluate these domains we will assess: 1) Recruitment and retention of participants 2) Intervention adherence 3) Program satisfaction 4) The extent to which the intervention was delivered as planned (derived from the Fidelity Checklist [Appendix Z], described in the measures section).

Aim 2: To evaluate the preliminary efficacy of the CTSC and CTSC + AE, as compared to HE, on increasing biochemically verified 7-day point prevalence smoking abstinence smoking abstinence rates at 3 and 6 month follow-up in Latino smokers. We hypothesize that participants in the CTSC + AE group will demonstrate significantly greater rates of biochemically verified 7-day smoking point prevalence abstinence at 3 and 6-month follow-up

Aim 3: To evaluate the efficacy of the CTSC+AE condition, as compared to the HE and CTSC groups, on increasing adherence to nicotine patch treatment (NRT adherence is defined as the proportion of days of NRT use over the number of patches made available).

Aim 4 (Exploratory): To examine potential mediators and moderators of the relationship between our primary outcomes (Aims 1-3) and our treatment groups. For this exploratory aim we will examine several variables that may influence treatment outcomes including gender, age, alcohol use, health related fatalism, sense of control and acculturation.

<u>Analytic and Design Considerations</u>

1) Latinos in the Houston Metropolitan area are heterogeneous with respect to country of origin (or national identity). We recognize the challenge of developing an intervention that adequately accounts for differences associated with this heterogeneity. We considered focusing on one or two groups e.g., Mexicans & Mexican Americans. However, we selected to focus on various national origins in order to take advantage of the unique opportunity for developing an intervention with the greatest potential for generalizability to Latinos of all origins. Houston is an ideal geographic locale for conducting this research since Latino subgroups are well represented in the population. In one of our previous studies (*Project Impact 1)*, Latinos across national origin groups were found to be similar on key demographic and smoking related variables including. Also, investigators have previously identified values and characteristics that are shared among Latino sub-groups in the U.S., providing a rationale for developing an intervention relevant to multiple Latino subgroups[32].

2) Despite our randomized design, it is possible that baseline participant characteristics that are potential confounders may be unevenly distributed. This potential problem will be closely monitored and adjustments to the statistical analysis plan will be made if

necessary.

3) Our primary research question compares the three treatment groups (CTSC, CTSC+AE, & HE) on the mean percentage of days of NRT adherence during the 12 weeks of treatment. In our preliminary study of Latino smokers (*Project Impact 1*) we found that on average participants were adherent on 46.7% (SD=36) of days during the 12 weeks of NRT treatment. Based on this finding, we estimate that our control condition (HE) will show a similar rate of NRT adherence (adherent on 56% of treatment days). We are guided primarily by our expectations that clinically significant improvements in outcomes will be achieved with >75% adherence and we base our estimate of the NRT adherence rate for our CTSC+AE condition on two studies reporting NRT adherence rates for interventions that sought to increase adherence but did not exclusively target adherence. The adherence rates of the experimental condition in these studies ranged from 88-93%. The current study is not only designed to test the feasibility of CTSC and CTSC+AE (Aim 1), but to evaluate the CTSC+AE intervention compared to HE and CTSC groups. It is not expected to be a definitive efficacy trial and the proposed sample will yield power to detect only large effects. Due to the limited scope of this development project, this study will not be powered for formal hypothesis testing of group differences. Nevertheless, the proposed study will conduct analyses investigating distributional assumptions and co-variation among examined predictors which will inform the design of a future efficacy trial that will be fully powered.

4) Other smoked non-tobacco substances can potentially interfere with our carbon monoxide testing. To address this concern, prior to all CO testing, participants will be asked whether they have smoked non-tobacco substances in the last two days. If so, the participant will be asked to provide an additional CO sample after 2 days of non-tobacco substance abstinence.

5) The two treatment interventions are not completely time-matched and participants in the CTSC+AE condition will receive a slightly greater dosage of the behavioral intervention than CTSC and HE. Therefore, dosage effects may confound our results. In designing our study, we considered this limitation. However, we made a decision to prioritize a design where the control condition (HE) reflects a minimum standard of care for individuals undergoing NRT treatment so that we can assess the real-world clinical implications of our intervention.

6) Missing Data: An intent-to-treat analysis will be utilized to account for missing data. We will assume non-adherence and/or smoking relapse on days for which we do not have data. Exhaustive efforts will be made to schedule visits so as to minimize such instances.

12.0 Data and Protocol Management

Data Safety and Monitoring by Study Personnel

We have developed a data and safety monitoring plan for this project that is

commensurate with the risks posed by the project. Data and safety monitoring for this project will consist of ongoing monitoring by the PI and research personnel. The plan includes procedures for:

1.Monitoring the progress of the trial and safety of the participants

2.Assuring compliance with the requirements for reporting of adverse events (AEs)

3.Assuring that any action resulting in a temporary or permanent suspension of a study is reported to the NIH official who is responsible for the grant

4.Ensuring data accuracy and protocol compliance.

The procedures for monitoring progress, safety, reporting of AEs, and assuring that actions resulting in suspension of the study are reported to the NIH are described in the sections below under *IRB Monitoring* and the *Guidelines for Filing Reports of Adverse Experiences at MDACC.* Plans and procedures for ensuring data quality and integrity are described in the final section entitled *Data Quality and Integrity*.

Study data will be managed using REDCap (Research Electronic Data Capture) electronic data capture tools hosted at MD Anderson. REDCap (www.project-redcap.org) is a secure, web-based application with controlled access designed to support data capture for research studies, providing: 1) an intuitive interface for validated data entry; 2) audit trails for tracking data manipulation and export procedures; 3) automated export procedures for seamless downloads to common statistical packages; and 4) procedures for importing data from external sources. REDCap (https://redcap.mdanderson.org) is hosted on a secure server by MD Anderson Cancer Center's Department of Research Information Systems & Technology Services.

REDCap has undergone a Governance Risk & Compliance Assessment (05/14/14) by MD Anderson's Information Security Office and found to be compliant with HIPAA, Texas Administrative Codes 202-203, University of Texas Policy 165, federal regulations outlined in 21CFR Part 11, and UTMDACC Institutional Policy #ADM0335. Those having access to the data file include the study PI and research team personnel. Users are authenticated against MDACC's Active Directory system. External collaborators are given access to projects once approved by the project sponsor. The application is accessed through Secure Socket Layer (SSL). All protected health information (PHI) will be removed from the data when it is exported from REDCap for analysis. All dates for a given participant will be shifted by a randomly generated number between 0 and 364, thus preserving the distance between dates. Dates for each participant will be shifted by a different randomly generated number. Following publication study data will be archived in REDCap.

Human Subjects Involvement and Characteristics Participants will be 36 Latino smokers at least 18 years of age that are interested and motivated to quit smoking. The

following inclusion criteria will be used: 1) Adults at least 18 years of age; 2) Current smoker (>5 cigarettes per day for the past 3 months) 3) Able to speak and read English or Spanish 4) Agree to participate in the study and be available for 6 weeks of treatment and 6 months of follow-up 5) Willing to set a quit date within 2 weeks of enrollment date 6) Identify as being of Latino heritage, ethnicity, or ancestry. Exclusion Criteria includes: 1) Individuals suffering from any unstable medical condition precluding the use of NRT 2) Currently using smokeless tobacco, electronic nicotine delivery systems (ENDS), nicotine replacement therapy, or other smoking cessation treatment 3) Pregnant or nursing 4) Suffering from a severe psychiatric disorder (assessed using self-reporting history of psychiatric diagnosis during the phone screening) that would interfere with participation 5) Diagnosis of substance dependence other than nicotine, screened using the Diagnostic and Statistical Manual of Mental Disorders, Fourth Edition (DSM IV TR) criteria 6) Individuals that do not have access to a working telephone.

Sources of Materials Data will be obtained through self-report questionnaires, interview questionnaires, and intervention session audio recordings. In addition, participants will be administered a test of expired air carbon monoxide level by a trained research assistant.

Adverse Events Given the non-invasive, minimal risk nature of the proposed research, we anticipate that the types of adverse experiences that may occur, if any, will focus on concerns about the discomfort of nicotine withdrawal or possible distress associated with sensitive issues arising during data collection. The study includes procedures to minimize these risks.

Adverse Experiences Associated with Nicotine Abstinence/Withdrawal: Participants may experience nicotine abstinence/withdrawal effects. These effects may include irritability, difficulty concentrating, insomnia, anxiety, dysphoria, and increased hunger. None of these effects result in serious adverse health consequences.

Adverse Experiences Associated with Self-Report: This study will also involve the use of questionnaires and interviews that could reveal sensitive information (e.g., assessment of mood disturbance). The instruments are not used to communicate psychiatric diagnosis to the participant and procedures are in place to protect participant confidentiality. However, if a mood disorder is suspected, the participant may be contacted. Possible outcomes of this contact could include referral to the participant's primary care physician and/or other

physician, and/or other mental health providers.

Adverse Experiences Associated with Nicotine Replacement Therapy (NRT) Patch The nicotine patch may cause nausea and/or headache. Participants may feel dizzy and/or light-headed, may be moody and/or have trouble sleeping. Participants may also have an allergic reaction to the patch that results in red, itchy, and/or sore skin around the patch.

Adverse Experiences Requiring Immediate Reporting Two types of Adverse Experiences require prompt reporting to the Institutional Review Board (IRB) and the study sponsor: Serious Adverse Experiences and Unexpected Adverse Experiences. For serious or unexpected AEs, a written report is submitted to the Office of Protocol Research (OPR) within 10 working days of the adverse experience. Unexpected fatal or life-threatening experiences are telephoned immediately to the OPR and the OPR will notify the appropriate sponsors within 3 days. For AEs that are not serious or not unexpected, written reports are submitted to OPR every month.

Serious Adverse Experiences For the majority of trials performed at MDACC, a serious adverse experience is a clinical event occurring subsequent to the administration of an agent or intervention which can be characterized as fatal, life-threatening, permanently disabling, requiring hospitalization, or an overdose.

Unexpected Adverse Experience An unexpected adverse experience is a clinical event that is not identified in nature, severity, or frequency in the investigator's brochure, protocol, or other pertinent supporting literature. At times, the occurrence of an unexpected adverse experience might not be suspected until more than one event has occurred. Once the clinical event has been identified, all cases should be reported.

Informed Consent Those who agree to participant will give informed consent. A written consent form will be presented and read to the potential participant, and an opportunity will be provided to review the form and to have any questions answered before the form is signed and filed. Consent forms contain a description of all procedures involved in the study, the risks and benefits

associated with the research, the rights of the participant (e.g. right to withdraw) and the potential limits of confidentiality (e.g. assessed suicidal or homicidal risk).

Data Quality and Integrity Due to the ongoing monitoring of the project, study investigators and staff will maintain quality assurance procedures for all data. Several procedures will be used to maintain the integrity of the data. All databases will be stored in a centralized location on one of the departmental servers, which is backed up daily, with access limited to specific users at the discretion of the PI. Hard copy data sources will be stored under lock and key in the Department of Health Disparities Research. Electronic data will be maintained by numerically coding of all data and by password-protecting computer files. Data will be stored on a secure server with a firewall to protect the data and to prevent unauthorized access. Databases on the server will be password protected and will be accessed only by research staff members who have met the MDACC IRB's requirement for training in Human Subjects Protections. Data transmissions to and from the data server will be encrypted. All participants will be assigned a unique identification code that will be stored separately from identifying information. Audio recordings of visits will be collected using a digital recording device (DRD). Once an interview session is completed a research assistant under the supervision of Dr. de Dios will immediately upload the audio recording to a secure departmental server. The DRD that was used for the interview will then be erased of the recording. Audio recordings on the secure server will be password protected and maintained and monitored on the secure server. Participants will be given full access to their data, if formally requested, after the completion of the study. Privacy safeguards will include appropriate password protection and physical security for all computer systems. Additional quality assurance procedures will include a data collection protocol as well as regularly scheduled meetings among the investigators and project staff to review problems and solutions, and discuss concerns. Data entry systems will specifically provide field checks, range checks for continuous variables, and valid value checks for categorical variables; checks for legitimate dates and times and logical consistency. Preliminary analyses will be initiated shortly after data collection begins to allow monitoring. All research staff involved in

the study will be carefully supervised by Dr. de Dios, and frequent team meetings will be held to assure that proper protocol is being followed.

13.0 Reporting Requirements

None.

14.0 Clinical Pharmacology

Pharmacotherapy: All participants (CTSC, CTSC+AE and HE conditions) will receive 12 weeks of NRT patch treatment - Nicoderm CQ (GlaxoSmithKline, Philadelphia, PA). Participants will be advised to use the patch according to the standard tapering schedule: 4 weeks of 21 mg, 4 weeks of 14mg, and 4 weeks of 7mg patches. Patch dispensation will occur at study visits. Participants receive only the number of patches necessary to last until the next study visit plus several extra patches should a patch fall off, become torn, etc., or should the visit be delayed. Based on our previous research, providing subjects with only enough patches to last until the subsequent visit improves compliance. We will carefully track pharmacotherapy use. Alongside the package insert included with the nicotine patches, participants will be given an instruction sheet on how to use the nicotine patch (Appendix Q).

15.0 References

1. Kaplan RC, Bangdiwala SI, Barnhart JM, et al. Smoking among U.S. Hispanic/Latino adults: the Hispanic community health study/study of Latinos. *Am J Prev Med.* 2014;46(5):496-506.
2. Centers for Disease Control (CDC) US Mortality Data: Division of Vital Statistics 2012. 2012; http://www.cdc.gov/nchs/data/nvsr/nvsr63/nvsr63_09.pdf.
3. Kang-Kim M, Betancourt JR, Ayanian JZ, Zaslavsky AM, Yucel RM, Weissman JS. Access to care and use of preventive services by Hispanics: state-based variations from 1991 to 2004. *Med Care.* 2008;46(5):507-515.
4. Guendelman S, Wagner TH. Health services utilization among Latinos and white non-Latinos: results from a national survey. *J Health Care Poor Underserved.* 2000;11(2):179-194.
5. Mayberry RM, Mili F, Ofili E. Racial and ethnic differences in access to medical care. *Med Care Res Rev.* 2000;57 Suppl 1:108-145.
6. Vargas Bustamante A, Chen J, Rodriguez HP, Rizzo JA, Ortega AN. Use of preventive care services among Latino subgroups. *Am J Prev Med.* 2010;38(6):610-619.
7. Arnett JJ. Emerging adulthood. A theory of development from the late teens through the twenties. *Am Psychol.* 2000;55(5):469-480.
8. Covey LS, Botello-Harbaum M, Glassman AH, et al. Smokers' response to combination bupropion, nicotine patch, and counseling treatment by race/ethnicity. *Ethn Dis.* 2008;18(1):59-64.
9. Gandhi KK, Foulds J, Steinberg MB, Lu SE, Williams JM. Lower quit rates

among African American and Latino menthol cigarette smokers at a tobacco treatment clinic. *Int J Clin Pract.* 2009;63(3):360-367.
10. Tobacco Use Among U.S. Racial/Ethnic Minority Groups: A Report of the Surgeon General. 1998; http://www.cdc.gov/tobacco/data_statistics/sgr/1998/complete_report/pdfs/complete_report.pdf.
11. Houston TK, Scarinci IC, Person SD, Greene PG. Patient smoking cessation advice by health care providers: the role of ethnicity, socioeconomic status, and health. *Am J Public Health.* 2005;95(6):1056-1061.
12. Denny CH, Serdula MK, Holtzman D, Nelson DE. Physician advice about smoking and drinking: are U.S. adults being informed? *Am J Prev Med.* 2003;24(1):71-74.
13. Lopez-Quintero C, Crum RM, Neumark YD. Racial/ethnic disparities in report of physician-provided smoking cessation advice: analysis of the 2000 National Health Interview Survey. *Am J Public Health.* 2006;96(12):2235-2239.
14. Reed MB, Burns DM. A population-based examination of racial and ethnic differences in receiving physicians' advice to quit smoking. *Nicotine Tob Res.* 2008;10(9):1487-1494.
15. Webb MS, Rodriguez-Esquivel D, Baker EA. Smoking cessation interventions among Hispanics in the United States: A systematic review and mini meta-analysis. *Am J Health Promot.* 2010;25(2):109-118.
16. de Dios MA, Anderson BJ, Stanton C, Audet DA, Stein M. Project Impact: a pharmacotherapy pilot trial investigating the abstinence and treatment adherence of Latino light smokers. *J Subst Abuse Treat.* 2012;43(3):322-330.
17. Cabriales JA, Cooper TV, Salgado-Garcia F, Naylor N, Gonzalez E. A randomized trial of a brief smoking cessation intervention in a light and intermittent Hispanic sample. *Exp Clin Psychopharmacol.* 2012;20(5):410-419.
18. Borrelli B, McQuaid EL, Novak SP, Hammond SK, Becker B. Motivating Latino caregivers of children with asthma to quit smoking: a randomized trial. *J Consult Clin Psychol.* 2010;78(1):34-43.
19. McAlister AL, Ramirez AG, Amezcua C, Pulley LV, Stern MP, Mercado S. Smoking cessation in Texas-Mexico border communities: a quasi-experimental panel study. *Am J Health Promot.* 1992;6(4):274-279.
20. Muñoz RF, Chen K, Bunge EL, Bravin JI, Shaughnessy EA, Pérez-Stable E. Reaching Spanish-speaking smokers online: a 10-year worldwide research program. *Rev Panam Salud Publica.* 2014;35(0):407-414.
21. Nevid JS, Javier RA. Preliminary investigation of a culturally specific smoking cessation intervention for Hispanic smokers. *Am J Health Promot.* 1997;11(3):198-207.
22. Woodruff SI, Talavera GA, Elder JP. Evaluation of a culturally appropriate smoking cessation intervention for Latinos. *Tob Control.* 2002;11(4):361-367.
23. Hill AL, Roe DJ, Taren DL, Muramoto MM, Leischow SJ. Efficacy of transdermal nicotine in reducing post-cessation weight gain in a Hispanic sample. *Nicotine Tob Res.* 2000;2(3):247-253.
24. Levinson AH, Borrayo EA, Espinoza P, Flores ET, Perez-Stable EJ. An exploration of Latino smokers and the use of pharmaceutical aids. *Am J Prev*

Med. 2006;31(2):167-171.
25. Levinson AH, Perez-Stable EJ, Espinoza P, Flores ET, Byers TE. Latinos report less use of pharmaceutical aids when trying to quit smoking. *Am J Prev Med.* 2004;26(2):105-111.
26. Shiffman S. Use of more nicotine lozenges leads to better success in quitting smoking. *Addiction.* 2007;102(5):809-814.
27. Shiffman S, Sweeney CT, Ferguson SG, Sembower MA, Gitchell JG. Relationship between adherence to daily nicotine patch use and treatment efficacy: secondary analysis of a 10-week randomized, double-blind, placebo-controlled clinical trial simulating over-the-counter use in adult smokers. *Clin Ther.* 2008;30(10):1852-1858.
28. Fish LJ, Peterson BL, Namenek Brouwer RJ, et al. Adherence to nicotine replacement therapy among pregnant smokers. *Nicotine Tob Res.* 2009;11(5):514-518.
29. Frankenfield DL, Wei, II, Anderson KK, Howell BL, Waldo D, Sekscenski E. Prescription medication cost-related non-adherence among Medicare CAHPS respondents: disparity by Hispanic ethnicity. *J Health Care Poor Underserved.* 2010;21(2):518-543.
30. Perez-Stable EJ, Marin BV, Marin G. A comprehensive smoking cessation program for the San Francisco Bay Area Latino community: Programa Latino Para Dejar de Fumar. *Am J Health Promot.* 1993;7(6):430-442, 475.
31. Marin G, Marin BV, Perez-Stable EJ, Sabogal F, Otero-Sabogal R. Changes in information as a function of a culturally appropriate smoking cessation community intervention for Hispanics. *Am J Community Psychol.* 1990;18(6):847-864.
32. Marin G, Perez-Stable EJ. Effectiveness of disseminating culturally appropriate smoking-cessation information: Programa Latino Para Dejar de Fumar. *J Natl Cancer Inst Monogr.* 1995(18):155-163.
33. Munoz RF, Marin BV, Posner SF, Perez-Stable EJ. Mood management mail intervention increases abstinence rates for Spanish-speaking Latino smokers. *Am J Community Psychol.* 1997;25(3):325-343.
34. Perez-Stable EJ SF, Marin G, Marin BV, Otero-Sabogal R. Evaluation of "Guia para Dejar de Fumar," a self-help guide in Spanish to quit smoking. *Public Health Rep.* 1991;106(05):564-570.
35. Wetter DW, Mazas C, Daza P, et al. Reaching and treating Spanish-speaking smokers through the National Cancer Institute's Cancer Information Service. A randomized controlled trial. *Cancer.* 2007;109(2 Suppl):406-413.
36. Stead LF, Perera R, Bullen C, et al. Nicotine replacement therapy for smoking cessation. *Cochrane Database Syst Rev.* 2012;11:CD000146.
37. Stead LF, Perera R, Bullen C, Mant D, Lancaster T. Nicotine replacement therapy for smoking cessation. *Cochrane Database Syst Rev.* 2008(1):CD000146.
38. Fiore MC. Treating tobacco use and dependence: an introduction to the US Public Health Service Clinical Practice Guideline. *Respir Care.* 2000;45(10):1196-1199.
39. Stanton CA, Lloyd-Richardson EE, Papandonatos GD, de Dios MA, Niaura R.

Mediators of the relationship between nicotine replacement therapy and smoking abstinence among people living with HIV/AIDS. *AIDS Educ Prev.* 2009;21(3 Suppl):65-80.
40. Stanton CA PG, Shuter J, Bicki A, Lloyd-Richardson EE, de Dios MA, Morrow K, Makgoeng S, Tashima KT, Niaura R. (In Press). . Outcomes of a Culturally Tailored Intervention for Cigarette Smoking Cessation among Latinos Living with HIV/AIDS. *Nicotine and Tobacco Research.* (IN PRESS).
41. Marin G, Marin, B. V. *Research with Hispanic Populations.* Newbury Park, CA: Sage; 1991.
42. de Dios M, Cano M, Childress S, Vaughan E, Wilson I, Niaura R. Agarrado por las ganas: A qualitative study of Latino smokers. 2015.
43. Annual Estimates of the Resident Population by Sex, Race, and Hispanic Origin for the

United States: April 1, 2000 to July 1, 2008. 2009.
44. Colby SL, Ortman JM. Projections of the Size and Composition of the U.S. Population: 2014 to 2060. *United States Census Bureau* 2015; P25-1143. Available at: https://www.census.gov/content/dam/Census/library/publications/2015/demo/p25-1143.pdf.
45. Fiore MC, Jaén CR, Baker TB, al. e. Treating Tobacco Use and Dependence: 2008 Update. . 2008; http://www.ncbi.nlm.nih.gov/books/NBK63952/.
46. Moore D, Aveyard P, Connock M, Wang D, Fry-Smith A, Barton P. Effectiveness and safety of nicotine replacement therapy assisted reduction to stop smoking: systematic review and meta-analysis. *BMJ.* 2009;338:b1024.
47. Silagy C, Lancaster T, Stead L, Mant D, Fowler G. Nicotine replacement therapy for smoking cessation. *Cochrane Database Syst Rev.* 2004(3):CD000146.
48. Silagy CA, Stead LF, Lancaster T. Use of systematic reviews in clinical practice guidelines: case study of smoking cessation. *BMJ.* 2001;323(7317):833-836.
49. Jackson PH, Stapleton JA, Russell MA, Merriman RJ. Nicotine gum use and outcome in a general practitioner intervention against smoking. *Addict Behav.* 1989;14(3):335-341.
50. Huang ES, Brown SE, Thakur N, et al. Racial/ethnic differences in concerns about current and future medications among patients with type 2 diabetes. *Diabetes Care.* 2009;32(2):311-316.
51. Perez-Stable EJ, Salazar R. Issues in achieving compliance with antihypertensive treatment in the Latino population. *Clin Cornerstone.* 2004;6(3):49-61; discussion 62-44.
52. Natarajan S, Santa Ana EJ, Liao Y, Lipsitz SR, McGee DL. Effect of treatment and adherence on ethnic differences in blood pressure control among adults with hypertension. *Ann Epidemiol.* 2009;19(3):172-179.
53. Oh DL, Sarafian F, Silvestre A, et al. Evaluation of adherence and factors affecting adherence to combination antiretroviral therapy among White, Hispanic, and Black men in the MACS Cohort. *J Acquir Immune Defic Syndr.* 2009;52(2):290-293.
54. Lanouette NM, Folsom DP, Sciolla A, Jeste DV. Psychotropic medication nonadherence among United States Latinos: a comprehensive literature review.

Psychiatr Serv. 2009;60(2):157-174.

55. Marin BV, Perez-Stable EJ, Marin G, Hauck WW. Effects of a community intervention to change smoking behavior among Hispanics. *Am J Prev Med.* 1994;10(6):340-347.
56. Fiore MC, Smith SS, Jorenby DE, Baker TB. The effectiveness of the nicotine patch for smoking cessation. A meta-analysis. *JAMA.* 1994;271(24):1940-1947.
57. Gourlay S. The pros and cons of transdermal nicotine therapy. *Medical Journal of Australia.* 1994;160(3):152-159.
58. Li Wan Po A. Transdermal nicotine in smoking cessation. A meta-analysis. *Eur J Clin Pharmacol.* 1993;45(6):519-528.
59. Tang JL, Law M, Wald N. How effective is nicotine replacement therapy in helping people to stop smoking? *BMJ.* 1994;308(6920):21-26.
60. Zimmerman M, Mattia JI. The Psychiatric Diagnostic Screening Questionnaire: development, reliability and validity. *Compr Psychiatry.* 2001;42(3):175-189.
61. Heatherton TF, Kozlowski LT, Frecker RC, Fagerstrom KO. The Fagerstrom Test for Nicotine Dependence: a revision of the Fagerstrom Tolerance Questionnaire. *Br J Addict.* 1991;86(9):1119-1127.
62. Becona E, Gomez-Duran B, Alvarez-Soto E, Garcia MP. Scores of Spanish smokers on Fagerstrom's Tolerance Questionnaire. *Psychol Rep.* 1992;71(3 Pt 2):1227-1233.
63. Hughes JR, Keely JP, Niaura RS, Ossip-Klein DJ, Richmond RL, Swan GE. Measures of abstinence in clinical trials: issues and recommendations. *Nicotine Tob Res.* 2003;5(1):13-25.
64. Cahalan D. *Drinking practices and problems: Research perspectives on remedial measures.* 1973.
65. Wolinsky FD, Wyrwich KW, Metz SM, Babu AN, Tierney WM, Kroenke K. Test-retest reliability of the Mirowsky-Ross 2 x 2 Index of the Sense of Control. *Psychol Rep.* 2004;94(2):725-732.
66. Franklin MD, Schlundt DG, Wallston KA. Development and validation of a religious health fatalism measure for the African-American faith community. *J Health Psychol.* 2008;13(3):323-335.
67. Hunter HL. *Ethnic Identity Development Measures: Bicultural Involvement Scale. Encyclopedia of Multicultural Psychology. SAGE Publications, Inc.* Thousand Oaks, CA: SAGE Publications, Inc.
68. Palmer KJ, Buckley MM, Faulds D. Transdermal Nicotine. A review of its pharmacodynamic and pharmacokinetic properties, and therapeutic efficacy as an aid to smoking cessation. *Drugs.* 1992;44(3):498-529.
69. Kreuter MW, Lukwago SN, Bucholtz RD, Clark EM, Sanders-Thompson V. Achieving cultural appropriateness in health promotion programs: targeted and tailored approaches. *Health education & behavior : the official publication of the Society for Public Health Education.* 2003;30(2):133-146.
70. Sobell L, Sobell M. Timeline Follow-Back. In: Litten R, Allen J, eds. *Measuring Alcohol Consumption*: Humana Press; 1992:41-72.
